# Supplementary material for: The Epithelial Sodium Channel—An Underestimated Drug Target
Source: Int J Mol Sci. 2023 Apr 24;24(9):7775. doi: 10.3390/ijms24097775 (PMC10178586; doi:10.3390/ijms24097775)
Supplement: Supplementary file 1 [file ijms-24-07775-s001.zip › Figures S1,S2,S3_17.04..pdf]

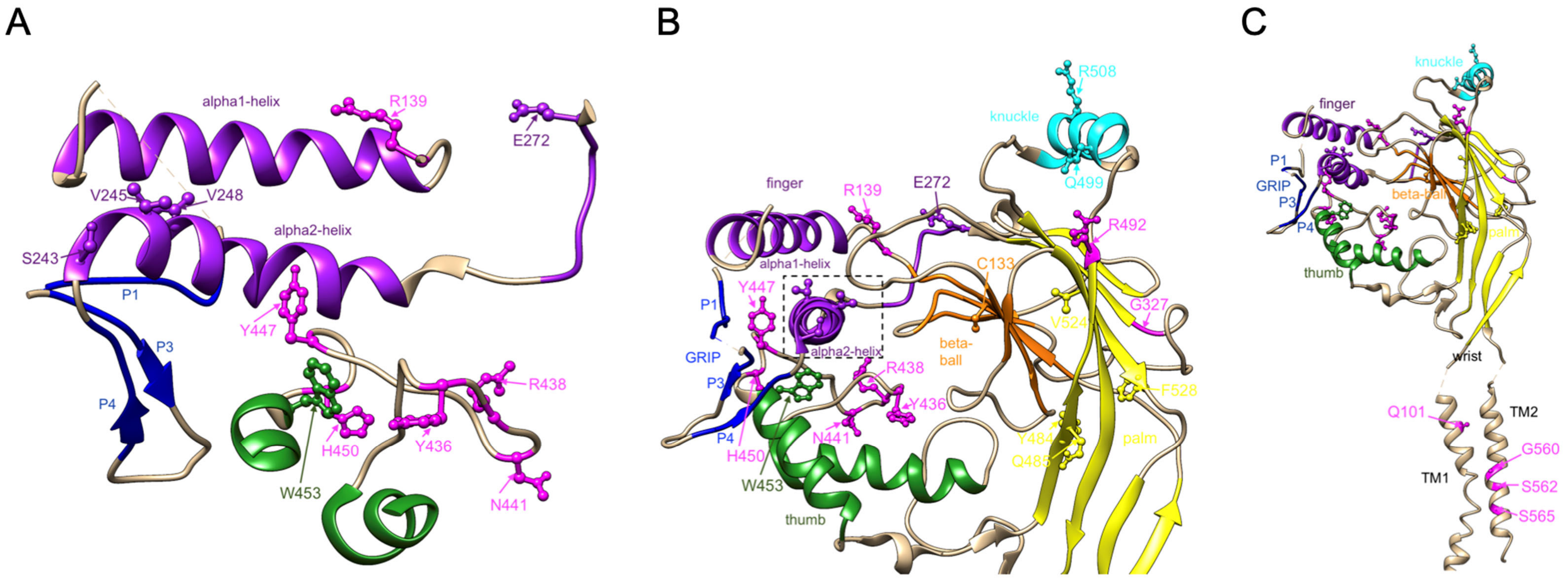

Figure S1. PHA-1B mutants mapped to extracellular loop and TM regions of α-ENaC. Position of PHA-1B mutants mapped to the structure PDB IB:6bqnA [38], the α-subunit of human ENaC. Cartoon representation with domains coloured as follows: knuckle, cyan; palm, yellow; finger, purple; gating release of inhibition of proteolysis (GRIP), blue; beta-ball, orange; thumb, green. Residues which are sites of PHA-1B-causing mutations have side chains rendered in ball and stick representation and are coloured according to the domain they occur in or magenta if located outside the defined domains. P1, P3 and P4 are strands constituting the GRIP domain; the inhibitory tract implicated in channel gating contains P1. **A.** α2-helix of the finger domain as depicted in B within the square with the dashed border, but rotated 90 degrees around y-axis in plane of paper to show side-on view of helix and location of residues which are sites of PHA-1B-causing mutations. **B.** Extracellular domain of α-ENaC showing position of PHA-1B mutants, except for those in the α2-helix for clarity. **C.** 6bqnA: human alpha ENaC subunit, showing extracellular domain and modelled TM regions (TM regions in 6bqn are not well resolved) with locations of mutations causing PHA-1B. Graphic created using UCSF Chimera, production version 1.16 (build 42360) [301].

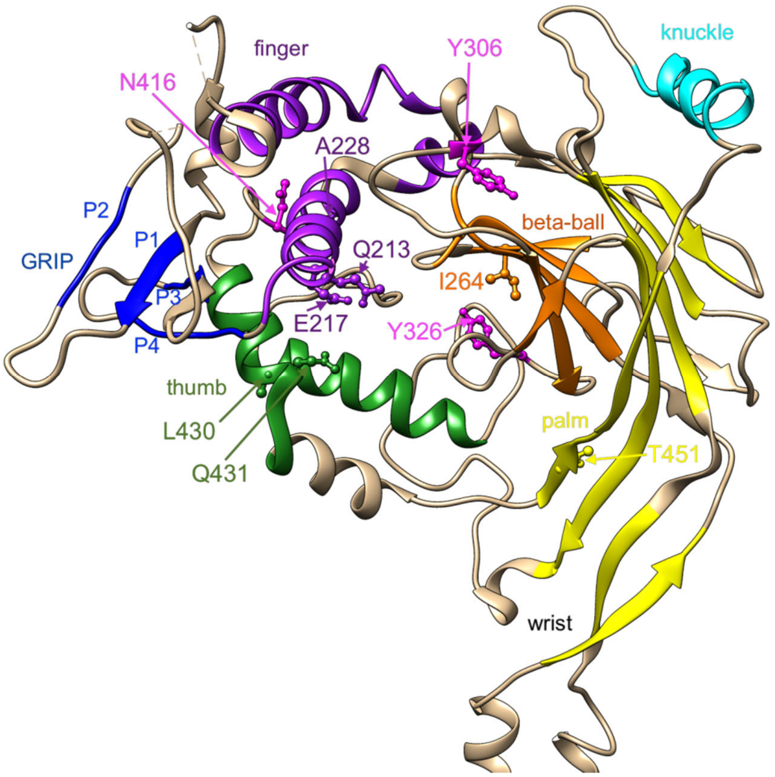

Figure S2. PHA-1B mutants mapped to extracellular loop of β-ENaC. Position of PHA-1B mutants mapped to the structure PDB IB:6bqnB [38], the beta subunit of human ENaC. Colouring and rendering as in Figure S1. P1, P2, P3 and P4 are strands constituting the GRIP domain, although the β-ENaC subunit is not cleaved by proteases. The TM region is not shown as there are no reported mutations causing PHA-1B in this region. Graphic created using UCSF Chimera, production version 1.16 (build 42360) [301].

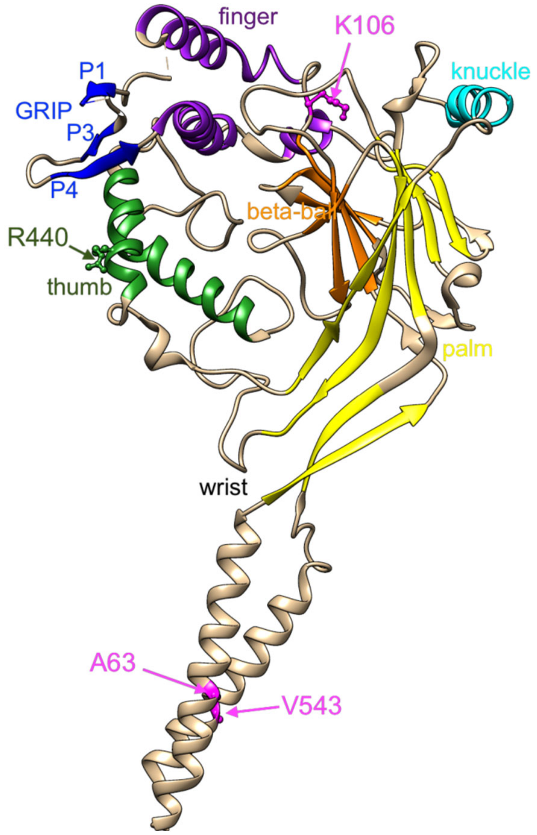

Figure S3. PHA-1B mutants mapped to extracellular loop and TM regions of γ-ENaC. Position of PHA-1B mutants mapped to the structure PDB IB:6bqnC [38], the γ-subunit of human ENaC. Colouring and rendering as in Supplementary Figure 1. P1, P3 and P4 are strands constituting the GRIP domain, the inhibitory tract implicated in channel gating contains P1. Predicted TM regions show locations of mutations causing PHA-1B (TM regions in 6bqn are not well resolved). Graphic created using UCSF Chimera, production version 1.16 (build 42360) [301].
