# Supplementary material for: The Epithelial Sodium Channel—An Underestimated Drug Target
Source: Int J Mol Sci. 2023 Apr 24;24(9):7775. doi: 10.3390/ijms24097775 (PMC10178586; doi:10.3390/ijms24097775)
Supplement: Supplementary file 1 [file ijms-24-07775-s001.zip › Tables S1,S2,S3_17.04..pdf]

Table S1. Mutations in *SCNN1A* (α-ENaC gene) which cause systemic pseudohypoaldosteronism (autosomal recessive PHA-1, PHA-1B).

Variant description NCBI NP_001029.2	DNA change (cDNA) NCBI RefSeq transcript NM_001038.6	Variant description NCBI RefSeqGene NG_011945.2 (GRCh38:12)	Exon	Variant description chromosomal GRCh38:12 NCBI NC_000012.12	dbSNP variant accession number	Reference
p.(Arg56*)	c.166C>T	g.7740C>T	2	g.6374618G>A	rs778872550	[272]
p.(Cys63*)	c.189C>A	g.7763C>A	2	g.6374595G>T		[273]
p.(Ile68Thrfs*76)	c.203_204del	g.7777_7778del	2	g.6374580_6374581del	rs765835593	[135]
p.(His69Arg)	c.206A>G	g.7780A>G	2	g.6374578T>C		[274]
p.(Arg73Cys)	c.217C>T	g.7791C>T	2	g.6374567G>A	rs763345732	[275]
p.(Gln101Lys)	c.301C>A	g.7875C>A	2	g.6374483G>T		[152]
p.(Cys133Tyr)	c.398G>A	g.7972G>A	2	g.6374386C>T		[276]
p.(Arg139Lys)	c.416G>A	g.7990G>A	2	g.6374368C>T	rs370256768	[277]
p.(Thr169Serfs*36)	c.505_506del	g.18736_18737del	3	g.6363622_6363623del	rs1014590535	[272]
p.(Pro197Alafs*9)	c.588dup	g.18819dup	3	g.6363539dup		[273]
p.(Ala200Glyfs*6)	c.598dup	g.18829dup	3	g.6363532dup	rs759611286	[274]
p.?	c.684+2T>A	g.18917T>A		g.6363441A>T		[278]
p.?	c.685-1G>A	g.20116G>A		g.6362242C>T		[145]
p.(Ser243Pro)	c.727T>C	g.20159T>C	4	g.6362199A>G	rs776069930	[269]
p.(Val245Trpfs*4)	c.729delA	g.20161del	4	g.6362197del	rs1592074026	[279]
p.(Val245Glyfs*65)	c.729-730delAG	g.20161_20162del	4	g.6363196_6363197del		[280]
p.(Val248*)	c.742del	g.20174del	4	g.6362184del		[273]
p.(Glu272Glyfs*39)	c.814dup	g.20246dup	4	g.6362112dup	rs747904876	[281]
p.?	c.875+2dup	g.20309dup		g.6362049dup		[282]
p.?	c.876+2delGAGT	g.20310del		g.6362048del		[283]
p.(Gly327Cys)	c.979G>T	g.26581G>T	5	g.6355777C>A	rs974854786	[284]
p.(Tyr436Ilefs*46)	c.1305del	g.27865del	8	g.6354493del	rs758014063	[272]
p.(Arg438Glyfs*44)	c.1311del	g.27871del	8	g.6354487del		[281]
p.(Asn441Thrfs*41)	c.1322_1322delA	g.27882del	8	g.6354477del		[283]

Variant description NCBI NP_001029.2	DNA change (cDNA) NCBI RefSeq transcript NM_001038.6	Variant description NCBI RefSeqGene NG_011945.2 (GRCh38:12)	Exon	Variant description chromosomal GRCh38:12 NCBI NC_000012.12	dbSNP variant accession number	Reference
p.(Tyr447Leufs*13)	c.1339dup	g.27899dup	8	g.6354459dup	rs754323537	[285]
p.(His450Lysfs*11)	c.1344_1347dup	g.27904_27907dup	8	g.6354453_6354456dup		[273] reported:c.1342_1343insTACA
p.(Trp453Glyfs*29)	c.1356del	g.27916del	8	g.6354443del		[284]
p.?	c.1360+1G>T	g.27921G>T		g.6354437C>A	rs573376286	[277]
p.?	c.1361-2A>G	g.32951A>G		g.6349407T>C		[273]
p.?	c.1439+1G>C	g.33032G>C		g.6349326C>G	rs1369791519	[281]
p.(Tyr484Thrfs*13)	c.1449del	g.33146del	10	g.6349212del	rs756434927	[279]
p.(Gln485*)	c.1453C>T	g.33150C>T	10	g.6349208G>A		[283]
p.(Arg492*)	c.1474C>T	g.33171C>T	10	g.6349187G>A	rs775543049	[276]
p.(Gln499Arg)	c.1496A>G	g.33193A>G	10	g.6349165T>C		[283]
p.(Arg508*)	c.1522C>T	g.33377C>T	11	g.6348981G>A	rs137852634	[135]
p.(Val524Ala)	c.1571T>C (c.1640T>C NM_0011589575.1)	Not available for NM_0011589575.1	12	g.6348785A>G (NM_0011589575.1)		[275] reported: V547A
p.(Phe528del)	c.1582_1584del	g.33584_33586del	12	g.6348777_6348779del	rs61759913	[286]
p.(Gly560Ser)	c.1678G>A	g.34153G>A	13	g.6348205C>T	rs772866436	[287]
p.(Ser562Pro)	c.1684T>C	g.34159T>C	13	g.6348199A>G		[288]
p.(Ser562Leu)	c.1685C>T	g.34160C>T	13	g.6348198G>A	rs137852635	[279]
p.(Ser565Tyr)	c.1694C>A	g.34169C>A	13	g.6348189G>T		[283]

Table S2. Mutations in *SCNN1B* (β-ENaC gene) which cause systemic pseudohypoaldosteronism (autosomal recessive PHA-1, PHA-1B).

Variant description protein NCBI NP_000327.2	DNA change (cDNA) RefSeq transcript NCBI NM_000336.3	Variant description RefSeqGene NCBI NG_011908.2 (GRCh38:16)	Exon	Variant description chromosomal GRCh38:16 NCBI NC_000016.10	Variant accession number (dbSNP)	Reference
na	17bp frameshift mutation in exon2	na	2	na		[275]
p.(Tyr29*)	c.87C>A	g.51417C>A	2	g.23348686C>A		[274]
p.(Gly37Ser)	c.109G>A	g.51439G>A	2	g.23348708G>A	rs137852706	[135]
p.(Leu174Tyrfs*12)	c.520_521insA	g.55740_55741insA	3	g.23353009_23353010insA		[272]
p.?	c.585+1G>A	g.55806G>A		g.23353075G>A	rs1411771150	[289]
p.(Gln213*)	c.637C>T	g.58081C>T	4	g.23355350C>T		[290]
p.(Glu217Argfs*38)	c.648dup	g.58092dup	4	g.23355361dup	rs747116196	[291]
p.(Ala228Hisfs*8)	c.682del		4	g.23355395del		[292]
p.(Ile264Serfs*16)	c.789del	g.70599del	5	g.23367868del		[272]
p.(Tyr306Thrfs*13)	c.915del	g.74064del	6	g.23371333del	rs1275275977	[291]
p.(Tyr326*)	c.978C>A		6	g.23371396C>A		[293]
p.(Asn416Glnfs*35)	c.1245dup	g.78561dup	8	g.23375830dup		[294]
p.?	c.1271-1G>C	g.79895G>C		g.23377164G>C		[295]
p.(Leu430Tyrfs*3)	c.1288del	g.79913del	9	g.23377182del		[296]
p.(Gln431Argfs*2)	c.1290delA		9	g.23377184del		[146]
p.?	c.1346+1G>A	g.79972G>A		g.23377241G>A		[274]
p.(Thr451Aspfs*6)	c.1348_1361del (c.1350_1363del)		10	g.23377332_23377345del		[146]
p.?	c.1466+1G>A	g.81499G>A	11	g.23378768G>A	rs1290855631	[296]
p.?	c.1542+1G>A	g.82901G>A	12	g.23380170G>A	rs550424284	[284]<br>[285]
p.(Asp546Asn)	c.1636G>A	g.83245G>A	13	g.23380514G>A	rs112069765	[151]
	>1300 bp deletion					[297]

Table S3. Mutations in *SCNN1G* (γ-ENaC gene) which cause systemic pseudohypoaldosteronism (autosomal recessive PHA-1, PHA-1B).

Variant description protein NCBI NP_001030.2	DNA change (cDNA) RefSeq transcript NCBI NM_001039.4	Variant description RefSeqGene NCBI NG_011909 .1 (GRCh38:16)	Exon	Variant description chromosomal GRCh38:16 NCBI NC_000016.10	Variant accession number (dbSNP)	Reference
p.(Leu34_Thr36del)	c.109_114del		2	g.23186380_23186385del		[298] reported: c.102_107del
p.(Ala63Pro)	c.187G>C	g.8740G>C	2	g.23186458G>C		[286]
p.(Lys106_Ser108delinsAsn)	c.318 -1G>A	g.11652G>A		g.23189370G>A	rs1567262640	[299]
p.(Thr176Argfs*9)	c.527_528delCA	g.11862_11863del	3	g.23189580_23189581del		[283]
p.(Arg440*)	c.1318C>T	g.34983C>T	9	g.23212701C>T		[290]
p.?	c.1570 -1G>A	g.37370G>A		g.23215088G>A	rs1596779402	[300]
p.(Val543Leufs*56)	c.1626del	g.37427del	13	g.23215145del	rs1596779433	[300]
